# Azeliragon ameliorates Alzheimer's disease via the Janus tyrosine kinase and signal transducer and activator of transcription signaling pathway

**DOI:** 10.6061/clinics/2021/e2348

**Published:** 2021-03-01

**Authors:** Lijuan Yang, Yepei Liu, Yuanyuan Wang, Junsheng Li, Na Liu

**Affiliations:** I Nursing Faculty of Xingtai Medical College, Xingtai, Hebei 054008, China; II Medical Image Center, Xingtai City Fifth Hospital, Xingtai, Hebei 054008, China

**Keywords:** Alzheimer's Disease, NLRP1, TTP488, JAK/STAT Signaling Pathway

## Abstract

**OBJECTIVES:**

TTP488, an antagonist of the receptor for advanced glycation end-products, was evaluated as a potential treatment for patients with mild-to-moderate Alzheimer’s disease (AD). However, the mechanism underlying the protective action of TTP488 against AD has not yet been fully explored.

**METHODS:**

Healthy male rats were exposed to aberrant amyloid β (Aβ) 1-42. Lipopolysaccharide (LPS) and the NOD-like receptor family pyrin domain containing 1 (NLRP1) overexpression lentivirus were injected to activate the NLRP1 inflammasome and exacerbate AD. TTP488 was administered to reverse AD injury. Finally, tofacitinib and fludarabine were used to inhibit the activity of Janus tyrosine kinase (JAK) and signal transducer and activator of transcription (STAT) to prove the relationship between the JAK/STAT signaling pathway and TTP488.

**RESULTS:**

LPS and NLRP1 overexpression significantly increased the NLRP1 levels, reduced neurological function, and aggravated neuronal damage, as demonstrated by the impact latency time of, time spent by, and length of the platform covered by, the mice in the Morris water maze assay, Nissl staining, and immunofluorescence staining in rats with AD.

**CONCLUSIONS:**

TTP488 administration successfully reduced AD injury and reversed the aforementioned processes. Additionally, tofacitinib and fludarabine administration could further reverse AD injury after the TTP488 intervention. These results suggest a new potential mechanism underlying the TTP488-mediated alleviation of AD injury.

## INTRODUCTION

Alzheimer’s disease (AD), largely considered to be caused by aberrant amyloid β (Aβ) accumulation and the most common cause of dementia, is clinically characterized by a progressive and irreversible loss of cognitive functions and is pathologically characterized by the loss of synapses and neuronal death (1,2). Aβ is known to bind to the receptor for advanced glycation end-products (RAGE), an immunoglobulin supergene family member expressed on multiple cell types in the brain and periphery, which leads to sustained inflammatory states that play a crucial role in AD (3,4).

Neuroinflammation plays an important role in the development and progression of AD. The inflammatory process in AD is driven by activated microglia and astrocytes, which induce pro-inflammatory response factors and related signaling pathways, resulting in synapse and neuron injury and further activation of inflammatory cells (5,6).

The NOD-like receptor family pyrin domain containing 1 (NLRP1), was the first member of the NLR family that is considered a critical component of the inflammasome and appears to be expressed ubiquitously (7). Recently, it has been reported that cerebellar granule neurons in rats subjected to oxygen and glucose deprivation could increase the NLRP1 mRNA levels and aggravate ischemic injury (8). Aβ and other abnormally aggregated proteins can induce the NLRP1-mediated activation of interleukin-1β (IL-1β) and IL-18, which promote the maturation and secretion of inflammatory factors to participate in the inflammatory response and represent a new mechanism underlying the pathogenesis of AD, which is “pyroptosis” (9–11). The Janus tyrosine kinase (JAK) and the signal transducer and activator of transcription (STAT) (JAK/STAT) signaling pathway is involved in the inflammatory effect of inflammatory cytokines such as IL-1β (12). The JAK/STAT signaling pathway plays a vital role in the inflammation in AD and serves as an anti-inflammatory target in studies (13). The activation of NLRPs and subsequent IL-1β production by neutrophils could be blocked by tofacitinib, which interferes with the JAK/STAT signaling pathway (14). These studies indicated that the NLRP inflammasome may be related to the JAK/STAT signaling pathway.

TTP488 (synonyms: Azeliragon), which was discovered by TransTech Pharma, Inc., serves as an active, centrally acting antagonist of the RAGE-RAGE ligand interaction and has been gradually applied in clinics (15-17). Chronic administration of TTP488 in AD transgenic mice has been reported to reduce the amyloid load in the brain, ameliorate behavioral damage, and normalize electrophysiological recordings (17). However, the mechanism whereby TTP488 ameliorates AD injury has not yet been fully clarified.

In this study, we focused on the TTP488-mediated alleviation of AD injury, which occurs via the inhibition of NLRP1 inflammasome activation, using lipopolysaccharide (LPS) and an NLRP1 overexpression lentivirus (oeNLRP1) to suppress the effects of TTP488. Furthermore, we used tofacitinib and fludarabine to inhibit the progress of NLRP1 inflammasome activation. Our findings indicate that TTP488 possibly reverses AD by affecting NLRP1 inflammasome activation via the JAK/STAT signaling pathway. These results may provide a new strategy for AD development by inflammation and pyroptosis.

## MATERIALS AND METHODS

### Animals

Eight- to twelve-week-old healthy male Sprague-Dawley (SD) rats, supplied by Liaoning Changsheng Biotechnology Co., Ltd. (Shenyang, Liaoning, China), were housed in a temperature- and light-controlled environment under pathogen-free conditions and provided unlimited access to food and water (12/12h light/dark cycle, 60±5% humidity, and at 22±3°C) (18). All animals used in this experiment were cared for according to the Guide for the Care and Use of Laboratory Animals (NIH Publication No. 85-23, revised 1996).

### Aβ preparation

The dried synthetic Aβ1-42 peptide (Sigma-Aldrich, St. Louis, USA) was first dissolved in DMSO and then diluted in PBS to obtain a 250 mM stock solution. This solution was incubated at 4°C for at least 24h and stored at -80°C until use (15). Before use, the solution was centrifuged at 12000 *g* for 10 min, and the supernatant was used as an oligomeric Aβ sample. The reverse sequence peptide for Aβ1-42 prepared in the same way was used as a control.

### Rat model of AD

Rats were anesthetized by the intraperitoneal injection of 10% chloral hydrate (1 mL/100 g) and fixed in the stereotactic head frame after conventional skin preparation (19). The median line of the skull and anterior fontanelle was exposed. A burr hole was bored in the right and left lateral cerebral ventricles (anterior-posterior axis, 1.0 mm; mediolateral axis, 2.0 mm). A 10-μL microinjection pump was stereotactically inserted until a depth of 3.5 mm. Next, 5 μL of Aβ1-42 was injected into both sides. The rats in the sham group were administered 5 μL of Aβ42-1, as previously described (19).

### Reagents and antibodies

Tofacitinib (S5001, Selleck, Texas, USA), fludarabine (S1491, Selleck, Texas, USA), TUNEL reaction solution (ab66108, Abcam, Cambridge, UK), TTP488 (HY-50682, MedChem Express, Sollentuna, Sweden), LPS (L8880, Solarbio, Beijing, China), caspase-1 ELISA kits (KL15200, KALANG, Shanghai, China), IL-1β ELISA kits (RLB00, R&D Systems, Minneapolis, USA), IL-18 LISA kits (ab213909, Abcam, Cambridge, UK), Anti-NLRP1 antibody (ab3683, Abcam, Cambridge, UK), anti-NeuN antibody (ab177487, Abcam, Cambridge, UK), Anti-caspase-1 antibody (BM4291, Boster, Wuhan, China), Anti-IL-1β antibody (ab9722, Abcam, Cambridge, UK), Anti-IL-18 antibody (PB0058, Boster, Wuhan, China), anti-β-actin antibody (Boster, BA2305, Wuhan, China), followed by secondary antibodies conjugated to horseradish peroxidase anti-rabbit IgG (H+L) (AS014, ABclonal, Wuhan, China), and anti-mouse IgG (H+L) (AS003, ABclonal, Wuhan, China) were used in this study.

### Administration of oeNLRP1 and other chemicals

The oeNLRP1 (5′-AAGAAGGAGGAGCTGAAGGA-3′) and the control lentivirus (5′-TGTTCTCTGCCTGCCTGATA-3′) were designed and chemically synthesized by GenePharma Corporation, Shanghai, China. The lentivirus vectors were stored at −80°C. Stereotactic injection of lentivirus vectors was performed as described previously (20). After injection, the needle was kept at the site for 15 min and then slowly withdrawn. The animals were administered with Aβ1-42 after 15 days (20).

Intraperitoneal injection of LPS (0.1 mg/kg) was used to activate the NLRP3 inflammasome and exacerbate AD (21). Tofacitinib (20 mg/kg) (22) or fludarabine (20 mg/kg) (23) were used to inhibit the JAK and STAT signaling pathways.

### Morris water maze (MWM) assay

The MWM assay was conducted during the last 6 days of the treatment period. It was performed in a circular pool (diameter, 120 cm) filled with opaque water at a temperature of 22±1°C. The rats were allowed to search for the platform for 60s. If a rat failed to find the platform, it was then picked up and placed on the platform for 15s, as previously described (24). The behaviors of all rats’ were monitored using a video camera and analyzed using a computer-controlled system (Beijing Sunny Instruments Inc., Beijing, China).

### Western blot analysis

Equal amounts of protein samples (40 μg) from each brain sample were subjected to 12% SDS-polyacrylamide gel electrophoresis and transferred onto a nitrocellulose membrane (24). All data were obtained using the ChemiDoc^TM^ Touch Imaging System and analyzed with the Image Lab 3.0 software (Bio-Rad, California, USA).

### Immunofluorescence staining

Expression levels of NLRP1 and NeuN were detected by IF staining. A paraffin-embedded tissue section (5 mm thickness) was dewaxed using xylene and dehydrated using graded concentrations of alcohol and incubated with 3% H_2_O_2_ for inhibiting endogenous peroxidase activity (24). After blocking in 10% goat serum for 10 min at room temperature, the section was incubated with the primary antibodies in a blocking solution overnight at 4°C (24). Then, the slides were washed in PBS, followed by treatment with HRP-labeled anti-rabbit IgG (1:100; Beyotime, China) for 30 min at 37°C and washing with PBS. Images were acquired with a Nikon Eclipse Ni inverted microscope (TE2000, Nikon, Tokyo, Japan).

### Enzyme-linked immunosorbent assay (ELISA)

For the rat samples, caspase-1, IL-1β, and IL-18 levels were measured from the frontal cortex tissue extracted in a cell lysis buffer. Caspase-1, IL-1β, and IL-18 activity levels were assayed by ELISA kits according to the manufacturer’s instructions (24).

### Terminal deoxynucleotidyl transferase-mediated dUTP nick-end labelling (TUNEL) staining

The level of apoptosis in the tissue section was determined by TUNEL assay, according to the manufacturer’s instructions (23). Images were obtained by fluorescence microscopy at 400× magnification.

### Nissl staining

Neuronal morphology in brain sections was evaluated after the cresyl violet staining of Nissl bodies. Briefly, after being deparaffinized, the tissue sections were incubated with cresyl violet solution, destained in 96% ethanol containing 0.5% acetic acid, dehydrated with isopropanol, cleared in xylene, and mounted under coverslips (6). A computer-assisted light microscope (Olympus, BX51) was used to scan the sections. Densities of Nissl-positive cells were calculated using the Image-Pro Express software (Media Cybernetics, Silver Spring, MD, USA). The total cell counts were averaged from six sections per animal.

### Statistical analysis

Data were expressed as the means±standard error. Data statistical analysis and correlation analysis were performed using GraphPad Prism 6.0 (GraphPad Software, San Diego, USA). Survival analysis was performed using SPSS 19.0 (IBM, New York, USA). Differences were analyzed using one-way ANOVA, and multiple comparisons were analyzed using the Sidak test. All differences were considered statistically significant at a *p*<0.05.

## RESULTS

### AD was associated with NLRP1 inflammasome activation

**Figure 1:**
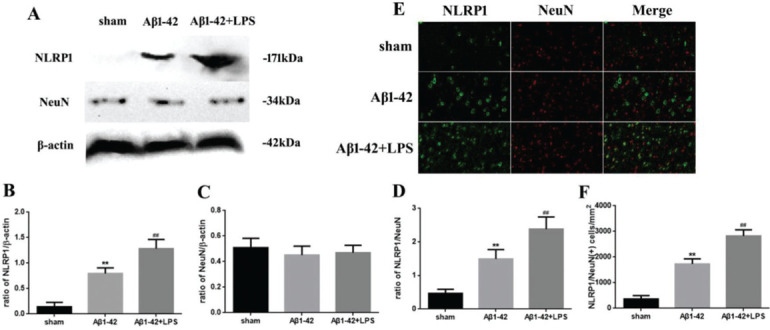
(A) Western blotting assay for detecting the expression of NLRP1 and NeuN. (B, C) Quantification of the expression of NLRP1 and NeuN. (D) NLRP1/NeuN ratio. (E) Immunofluorescence assay for detecting the expression of NLRP1 and NeuN (×400) and (F) NLRP1/NeuN (+) cells in samples from the sham, Aβ1-42, and Aβ1-42+LPS groups. Protein levels were normalized to those of β-actin (Aβ1-42 *vs*. sham group, **p<0.05; Aβ1-42+LPS *vs*. Aβ1-4 group, ^##^ *p*<0.05, n=6 per group. All data were represented as the mean±standard error).

The relationship between NLRP1 inflammasome activation and AD is shown in Figure 1. The NLRP1 expression in the Aβ1-42 group increased significantly (*p*<0.05) compared to that in the sham group. Moreover, in the Aβ1-42+LPS group, the NLRP1 expression was further increased compared to that in the Aβ1-42 group. Nevertheless, the NeuN expression did not change significantly (Figure 1A-C). The variation in the NLRP1/NeuN ratio was similar to that in the NLRP1 expression (Figure 1D). Immunofluorescence staining of NLRP1/NeuN is shown in Figure 1E. The number of NLRP1/NeuN-positive cells in the Aβ1-42 group increased significantly compared to that in case of the sham group. Similarly, in the Aβ1-42+LPS group, the number of NLRP1/NeuN-positive cells increased further, compared to that in case of the Aβ1-42 group (Figure 1F).

### TTP488 can reverse AD injury *in vivo*

**Figure 2:**
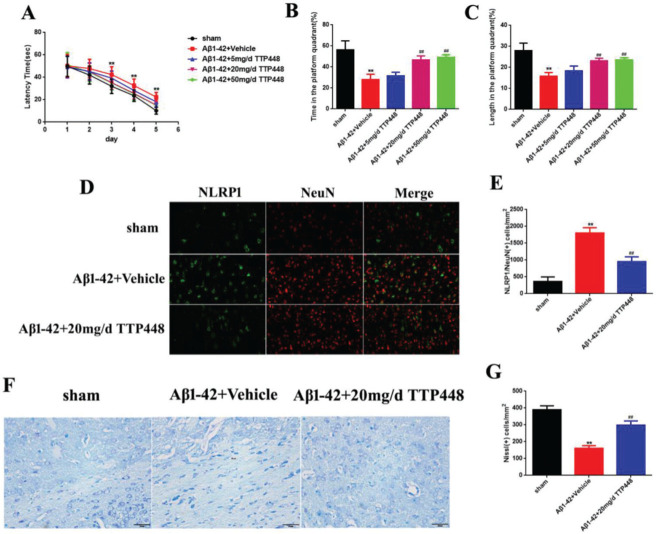
(A) The latency time of, (B) the time spent by, and (C) the length covered by, the mice from the sham, Aβ1-42+Vehicle, Aβ1-42+5 mg/d TTP488, Aβ1-42+20 mg/d TTP488, and Aβ1-42+50 mg/d TTP488 groups in the platform quadrant in the Morris water maze. (D) Immunofluorescence assay for detecting the expression of NLRP1 and NeuN (×400) and (E) NLRP1/NeuN (+) cells. (F) Nissl staining and (G) Nissl (+) cells in samples from the sham, Aβ1-42+Vehicle, and Aβ1-42+20 mg/d TTP488 groups (Aβ1-42+Vehicle *vs*. Sham group, **p<0.05; Aβ1-42+20 mg/d TTP488 *vs*. Aβ1-4+Vehicle group, ^##^ *p*<0.05, n=6 per group).

TTP488 is a RAGE antagonist with anti-inflammatory properties. In order to investigate the effect of TTP488 on AD, we designed three concentrations of TTP488 and verified their effects *in vivo* (Figure 2). The MWM results are shown in Figure 2A-C. The latency time was significantly extended in the Aβ1-42+vehicle group after 3 days, compared to that in the sham group. Furthermore, there was no statistical significance between the latency times in the Aβ1-42+vehicle and Aβ1-42+TTP488 groups (Figure 2A). The time and length in the platform quadrant were increased in the Aβ1-42+20 mg/d and 50 mg/d TTP488 groups, compared to those in the Aβ1-42+vehicle group (Figure 2B, C). Moreover, there was no difference between the effects of TTP488 at 20 mg/d and 50 mg/d; therefore, we used 20 mg/d as the optimum concentration of TTP488 for intervention in the follow-up experiments. The results of the immunofluorescence staining analysis for detecting NLRP1/NeuN is shown in Figure 2D; the number of NLRP1/NeuN-positive cells in the Aβ1-42+vehicle group increased significantly compared to that in the sham group. However, in the Aβ1-42+20 mg/d TTP488 group, the number of NLRP1/NeuN-positive cells decreased (Figure 2E). The results of the Nissl staining are shown in Figure 2F; the Nissl staining in the Aβ1-42+vehicle group showed the presence of a disorderly loose cytoplasm, edema, and neurocyte karyopyknosis, and the number of Nissl bodies was significantly reduced compared to the case in the sham group. In contrast, administering TTP488 ameliorated the neurocyte injury and increased the number of Nissl bodies (Figure 2G).

### TTP488 reverses AD injury by affecting NLRP1 inflammasome activation

**Figure 3:**
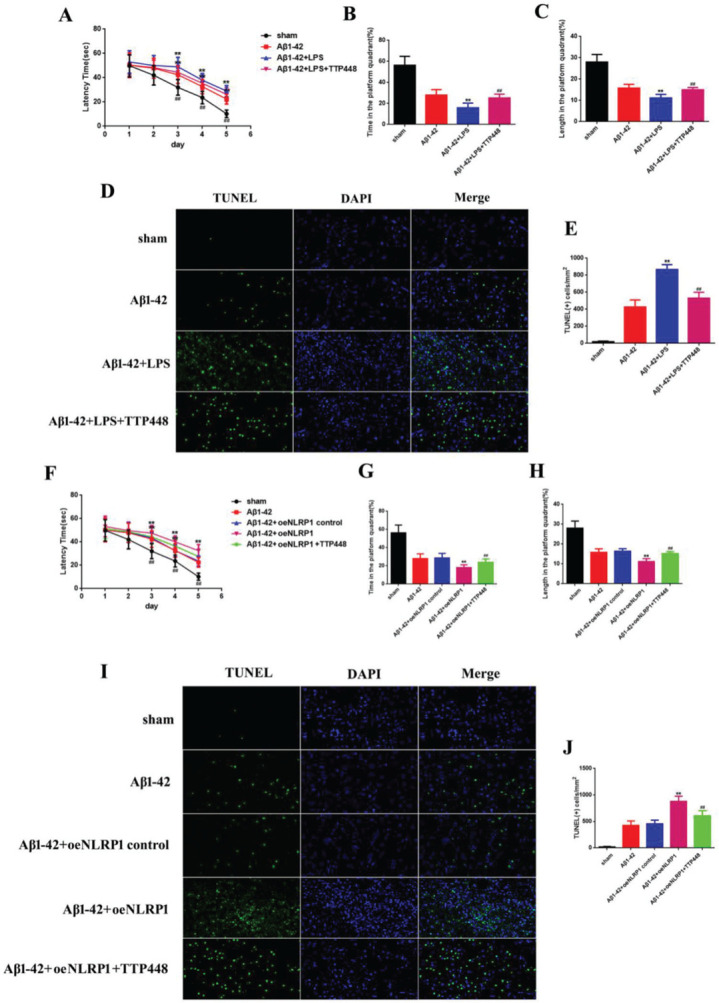
(A) The latency time of, (B) the time spent by, and (C) the length covered by, the mice in the platform quadrant in the Morris water maze. (D) TUNEL Immunofluorescence assay (×400). (E) TUNEL (+) cells in samples from the sham, Aβ1-42, Aβ1-42+LPS, and Aβ1-42+LPS+TTP488 groups. (F) The latency time, (G) the time, and (H) the length covered in the platform quadrant in the Morris water maze. (I) TUNEL immunofluorescence assay (×400) and (J) TUNEL (+) cells in samples from the sham, Aβ1-42, Aβ1-42+control oeNLRP1, Aβ1-42+oeNLRP1, and Aβ1-42+oeNLRP1+TTP488 groups (Aβ1-42+LPS or Aβ1-42+oeNLRP1 *vs*. Aβ1-42 group, **p<0.05; Aβ1-42+LPS+TTP488 or Aβ1-42+oeNLRP1+TTP488 *vs*. Aβ1-42+LPS or Aβ1-42+oeNLRP1 group, ^##^ *p*<0.05, n=6 per group).

As TTP488 could reverse AD injury, we explored whether this process occurred via NLRP1 inflammasome activation. We utilized LPS to activate the NLRP1 inflammasome (Figure 3). The MWM assay results are shown in Figure 3A-C; the latency time was significantly extended in the Aβ1-42+LPS group after 3 days, compared to the case for the Aβ1-42 group. Furthermore, the latency time after LPS-induced NLRP1 inflammasome activation was clearly shortened by administering TTP488 (Figure 2A). The time spent and length covered in the platform quadrant varied inversely with the latency time (Figure 3B, C). To investigate the effects of TTP488 on apoptosis after NLRP1 inflammasome activation, we used TUNEL staining to observe the changes (Figure 3D). The apoptosis level in the Aβ1-42+LPS group was increased compared with that in the Aβ1-42 group. The number of TUNEL-positive cells was also markedly reduced after TTP488 administration (Figure 2E). To further investigate the role of the NLRP1 inflammasome, we used the oeNLRP1 construct to induce the NLRP1 expression. The latency time was significantly extended in the Aβ1-42+oeNLRP1 group after 3 days, compared to that for the Aβ1-42 group. Furthermore, the latency time after oeNLRP1 administration was evidently shortened by administering TTP488 (Figure 2F). The time spent and the length covered in the platform quadrant varied inversely with the latency time (Figure 3G, H). Likewise, the apoptosis level in the Aβ1-42+oeNLRP1 group was higher than that in the Aβ1-42 group. The number of TUNEL-positive cells also reduced markedly after TTP488 administration (Figure 3I, J).

### TTP488 can reverse inflammation by affecting NLRP1 inflammasome activation in AD

**Figure 4:**
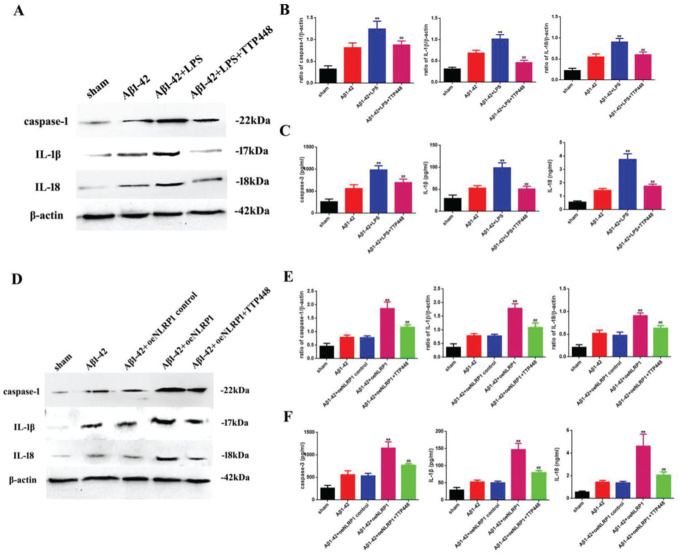
(A) Western blot assay for detecting the expression of caspase-1, IL-1β, and IL-18. (B) Quantification of the expression levels of caspase-1, IL-1β, and IL-18. (C) ELISA assay for quantifying the expression levels of caspase-1, IL-1β, and IL-18 in the sham, Aβ1-42, Aβ1-42+LPS, and Aβ1-42+LPS+TTP488 groups. (D) Western blot assay for detecting the expression of caspase-1, IL-1β, and IL-18. (E) Quantification of the expression levels of caspase-1, IL-1β, and IL-18. (F) ELISA assay for quantifying the expression levels of caspase-1, IL-1β, and IL-18 in the sham, Aβ1-42, Aβ1-42+control oeNLRP1, Aβ1-42+oeNLRP1, and Aβ1-42+oeNLRP1+TTP488 groups. Protein levels were normalized to those of β-actin (Aβ1-42+LPS or Aβ1-42+oeNLRP1 *vs*. Aβ1-42 group, **p<0.05; Aβ1-42+LPS+TTP488 or Aβ1-42+oeNLRP1+TTP488 *vs*. Aβ1-42+LPS or Aβ1-42+oeNLRP1 group, ^##^ *p*<0.05, n=6 per group).

As TTP488 could reverse AD injury by affecting NLRP1 inflammasome activation, we detected the inflammation level following TTP488 administration by western blotting and ELISA assays (Figure 4). In our experiments, we used LPS and the oeNLRP1 construct to activate NLRP1. As shown in Figure 4A, LPS boosted caspase-1, IL-1β, and IL-18 protein levels, compared to the case in the Aβ1-42 group, whereas caspase-1, IL-1β, and IL-18 protein levels were reduced by TTP488 (Figure 4B). The activities of caspase-1, IL-1β, and IL-18 were detected by ELISA; they increased following LPS treatment and decreased following TTP488 administration, similar to the case for their protein levels (Figure 4C). As shown in Figure 4D, oeNLRP1 enhanced the caspase-1, IL-1β, and IL-18 protein levels, while the caspase-1, IL-1β, and IL-18 protein levels were restored by TTP488 (Figure 4E). The activities of caspase-1, IL-1β, and IL-18 were uniformly increased by oeNLRP1 and decreased by TTP488 (Figure 4F).

### The TTP488-mediated alleviation of AD, which occurred via NLRP1, was regulated through the JAK/STAT signal pathway

To eliminate the influence of the JAK/STAT signaling pathway involved in the TTP488-mediated recovery of AD injury, which occurred via NLRP1 inflammasome activation, we used tofacitinib and fludarabine, which are specific inhibitors of JAK and STAT, respectively (Figure 5). As shown in Figure 5A, tofacitinib and fludarabine markedly shortened the latency time, compared to the case in the Aβ1-42+oeNLRP1+TTP488 group. The time spent and the length covered in the platform quadrant varied inversely with the latency time (Figure 5B, C). Furthermore, the protein levels (Figure 5E-G) and activities of caspase-1, IL-1β, and IL-18 (Figure 5H-J) were also reduced following the administration of tofacitinib and fludarabine.

## DISCUSSION

AD is a progressive disease mainly manifesting as progressive memory defects, gradual decline in language, spatial recognition, cognitive ability, independent living ability, and abnormal mental behavior; it seriously affects the physical health and quality of life of the elderly (25). Aβ could lead to sustained inflammatory states that play a crucial role in AD (2). In human AD patients, the levels of neuroinflammation markers, such as INF-gamma, TNF-alpha, and nitric oxide, were higher in mild and severe stages than in the earlier phases, indicating the progression of the disease (26). Neuroinflammation has been a target of drug therapy for AD treatment (27).

Inflammasomes are intracellular polymeric protein complexes composed of the NOD-like receptor family pyrin (NLRP), caspase-1, and apoptotic granular proteins with the CARD domain (28). Studies have reported that NLRP1 inflammation corpuscles induce inflammatory responses and pyroptosis, while the suppression of their activity alleviates intrinsic immune responses, neuronal death, and age-related cognitive impairment (29,30). More importantly, another study based on candidate genes found a link between the NLRP1 gene polymorphism and AD patients (31). In our study, we used LPS, an inflammatory agonist of AD, to activate the NLRP1 inflammasome, and the expression of NLRP1 was found to be significantly increased following LPS treatment in the western blotting and immunofluorescence staining assays. These results highlight that LPS induces AD via NLRP1 inflammasome activation.

TTP488 has been reported to serve as an antagonist of the RAGE-RAGE ligand interaction through various clinical trials and is applied for AD therapy (32); however, studies on TTP488 are relatively lacking, and the mechanisms whereby TTP488 ameliorates AD injury have not yet been fully clarified. Therefore, we screened the optimum concentration of TTP488 and evaluated the spatial memory, levels of inflammatory markers, and nerve cells in rats with AD. The results showed that TTP488 significantly improved the spatial memory of rats, reduced the NLRP1 levels, and decreased the neuronal cell damage. Furthermore, to detect the inhibition of NLRP1 inflammasome activation in TTP488, we used LPS and the oeNLRP1 construct, which reversed the effects of TTP488 in our study. Thus, these findings show that the anti-inflammatory function of TTP488 may be achieved via the inhibition of NLRP1 inflammasome activation.

Pyroptosis is characterized by cellular lysis and the release of cytosolic contents into the extracellular space; this is associated with NLRP1 inflammasome activation, leading to the generation of a functional caspase-1-containing inflammasome. Thus, pyroptosis contributes to the pathogenesis of AD (33). To verify the anti-inflammatory function of TTP488, we also detected the levels of caspase-1, IL-1β, and IL-18, which are pyroptosis factors. The results showed that TTP488 markedly decreased the expression and activities of caspase-1, IL-1β, and IL-1 by inhibiting NLRP1 inflammasome activation.

NLRP activation and subsequent IL-1β production by neutrophils could be blocked by tofacitinib, which interferes with the JAK/STAT signaling pathways (34). However, whether the anti-inflammatory function of TTP488 occurs via the JAK/STAT signaling pathway has not yet been reported. To address this issue, we used tofacitinib and fludarabine, which block JAK and STAT, respectively, after NLRP1 inflammasome activation in AD in rats. Our results showed that tofacitinib and fludarabine further improved the spatial memory and reduced the inflammatory levels of pyroptosis after NLRP1 inflammasome activation.

Our findings indicated that TTP488 significantly inhibited NLRP1 expression and activation during AD in rats by modulating the JAK/STAT signaling pathway, and then improved the spatial memory and reduced the inflammatory levels of pyroptosis. Taken together, these findings suggest the existence of a new potential mechanism underlying the TTP488-mediated alleviation of AD injury.

## AUTHOR CONTRIBUTIONS

Yang L and Liu Y designed the study and wrote the first draft, and contributed equally to the manuscript and should be considered as the same first author. Wang Y polished the first draft and confirmed the methodology and material parts. Li J and Liu N analyzed the data, wrote, and revised the manuscript. All of the authors have read and approved the final version of the manuscript.

## Figures and Tables

**Figure:**
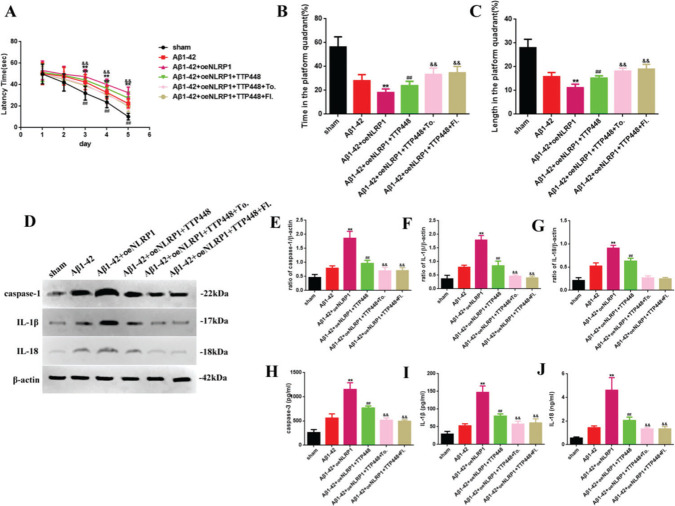
(A) The latency time of, (B) the time spent by, and (C) the length covered by, mice in the platform quadrant in the Morris water maze. (D) Western blot assay for detecting the expression of caspase-1, IL-1β, and IL-18. (E-G) Quantification of the levels of caspase-1, IL-1β, and IL-18. (H-J) ELISA assay for quantifying the expression levels of caspase-1, IL-1β, and IL-18 in the sham, Aβ1-42, Aβ1-42+oeNLRP1, Aβ1-42+oeNLRP1+TTP488, Aβ1-42+oeNLRP1+TTP488+Tofacitinib, and Aβ1-42+oeNLRP1+TTP488+Fludarabine groups. Protein levels were normalized to those of β-actin (Aβ1-42+oeNLRP1 *vs*. Aβ1-42 group, **p<0.05; Aβ1-42+oeNLRP1+TTP488 *vs*. Aβ1-42+oeNLRP1 group, ^##^ *p*<0.05; Aβ1-42+oeNLRP1+TTP488+Tofacitinib or Aβ1-42+oeNLRP1+TTP488+Fludarabine *vs*. Aβ1-42+oeNLRP1+TTP488 group, ^&&^ *p*<0.05, n=6 per group).
